# Survey of senescent cell markers with age in human tissues

**DOI:** 10.18632/aging.102903

**Published:** 2020-03-11

**Authors:** M. Laura Idda, Waverly G. McClusky, Valeria Lodde, Rachel Munk, Kotb Abdelmohsen, Martina Rossi, Myriam Gorospe

**Affiliations:** 1 Laboratory of Genetics and Genomics, National Institute on Aging Intramural Research Program, National Institutes of Health, Baltimore, MD 21224, USA; 2 Istituto di Ricerca Genetica e Biomedica, Consiglio Nazionale delle Ricerche, Sassari, Italy; 3 Department of Biomedical Sciences, University of Sassari, Sassari, Italy

**Keywords:** senescence in tissues, aging, p21, p16

## Abstract

Cellular senescence, triggered by sublethal damage, is characterized by indefinite growth arrest, altered gene expression patterns, and a senescence-associated secretory phenotype. While the accumulation of senescent cells during aging decreases tissue function and promotes many age-related diseases, at present there is no universal marker to detect senescent cells in tissues. Cyclin-dependent kinase inhibitors 2A (p16/CDKN2A) and 1A (p21/CDKN1A) can identify senescent cells, but few studies have examined the numbers of cells expressing these markers in different organs as a function of age. Here, we investigated systematically p16- and p21-positive cells in tissue arrays designed to include normal organs from persons across a broad spectrum of ages. Increased numbers of p21-positive and p16-positive cells with donor age were found in skin (epidermis), pancreas, and kidney, while p16-expressing cells increased in brain cortex, liver, spleen and intestine (colon), and p21-expressing cells increased in skin (dermis). The numbers of cells expressing p16 or p21 in lung did not change with age, and muscle did not appear to have p21- or p16-positive cells. In summary, different organs display different levels of the senescent proteins p16 and p21 as a function of age across the human life span.

## INTRODUCTION

Senescence is a cellular response to sublethal harm and developmental signals characterized by generally irreversible growth arrest, alterations in metabolic state, and changes in morphology and gene expression programs [1, 2]. Unlike other non-proliferative states such as quiescence and differentiation, senescence is associated with macromolecular damage and by changes in expressed proteins including elevations in cyclin-dependent kinase inhibitors p21 (CDKN1A) and p16 (CDKN2A), as well as the tumor suppressor p53 (TP53). Senescent cells also express and secrete factors that promote inflammation (e.g., the interleukins IL6, IL1B, and IL8), degrade the extracellular matrix (matrix metalloproteases, MMPs), and promote angiogenesis (e.g., vascular endothelial growth factor, VEGF); this trait is known as the senescence-associated secretory phenotype (SASP) [3, 4]. In addition to these features, senescent cells express a β-galactosidase (SA-β-gal) enzyme active at pH 6 that reflects a robust lysosomal activity [5, 6].

Senescent cells accumulate in tissues during aging and in a range of disease conditions [7, 8]. In addition to telomere shortening resulting from replicative exhaustion, various damaging stresses may lead to senescence, such as direct DNA damage from irradiation and chemicals, and mitochondrial dysfunction with increased production of reactive oxygen species (ROS) (reviewed in [1]).

Senescence is often viewed as a double-edged sword with both beneficial and detrimental effects [9]. Among its beneficial actions, senescence was shown to promote wound repair, developmental morphogenesis, and tumor suppression, mainly by triggering cell cycle arrest and the release of specific cytokines necessary for wound healing [10–14]. Among its detrimental actions, senescent cells contribute to chronic inflammation and tissue degeneration mainly derived from the production of the pro-inflammatory cytokines, growth factors, and MMPs that comprise the SASP. In this regard, the SASP was shown to alter tissue function and to accelerate the aging process by recruiting immune cells and extracellular matrix-remodeling complexes. Accordingly, in young individuals, senescence plays a key role in tumor surveillance and tissue repair, whereas in older individuals, the accumulation of senescent cells has been associated with tissue dysfunction and chronic conditions like cancer, cardiovascular disease and neurodegeneration [3, 9, 15, 16]. Importantly, clearance of senescent cells using genetic approaches or senolytic drugs has been shown to improve tissue function in different *in vivo* models of aging and age-associated diseases [17, 18]. For example, in mice, clearance of p16-positive senescent cells was shown to delay or prevent age-associated pathologies and losses such as sarcopenia, loss of adiposity, cataracts, cardiac hypertrophy, kidney disease, cancer, atherosclerosis, osteoarthritis, and neurodegeneration [19–26]. Similarly, elimination of senescent cells is found to be beneficial in a growing number of human pathologies, mainly cancer, but also cardiovascular disease, neurodegeneration, obesity, type 2 diabetes, sarcopenia, and osteoarthritis [23, 27, 28].

Our knowledge of aging biology and cell senescence has advanced remarkably in recent years, but the connection between senescence and aging is still poorly understood. At the same time, given the rising appreciation that senescent cells influence numerous physiological and pathological processes, there is strong interest in identifying and characterizing senescent cells in human tissues as a function of age. Detecting senescent cells *in vivo* has been challenging due to several major obstacles. Molecular markers of senescence are often inconclusive because they are also expressed in non-senescent cells in certain conditions, such as acute tissue damage. Similarly, SA-β-Gal positive staining is not exclusive of senescent cells, as it is detectable in non-senescent cells with high lysosomal activity. In addition, SA-β-Gal can only be detected in fresh tissues, thus limiting the analysis of archived tissues.

Despite these limitations, p16 protein and *p16* mRNA were previously described as markers of aging, as their levels are almost undetectable in healthy young tissues, but increase markedly during aging [29–32]. However, only a few studies have analyzed the accumulation of senescent cells in human tissues as a function of age. In this report, we describe the design of human tissue arrays using formalin-fixed, paraffin-embedded (FFPE) tissue sections spanning 10 major organs and three age groups – Young (13-35 years old), Middle-aged (40-59 years old), and Old (>65 years old) – followed by the systematic identification of cells positive for the senescent markers p16 and p21 as a function of age. Our analysis reveals specific patterns of distribution of cells expressing senescent markers in normal human tissues as a function of age.

## RESULTS AND DISCUSSION

### Changes in senescence marker proteins p16 and p21 in different organs

In order to catalog the abundance of cells expressing the senescence markers p16 and p21 in distinct age groups and tissues, we custom-designed tissue arrays (FFPE) comprising a panel of normal healthy tissues obtained from human donors of different ages (Array II, BioChain Institute; FDA 35, Pantomics, Inc.). The samples were grouped according to the ages of donors into Young, Middle-aged and Old as shown in Table 1. For each organ type and age group, the array included 5 individual tissue sections. To visualize the expression of p16 and p21, tissue arrays were probed with specific antibodies. The slides were then scanned and the acquired digital images were processed using a color deconvolution algorithm and were analyzed as explained in the Methods section. Cells with positive staining for p16 or p21 were counted in a selected area and then compared to the number of total cells in the same area. Negative control slides (incubated only with secondary antibody) were used for each staining (not shown). Representative micrographs for each tissue/organ and age group are shown: pancreas, kidney, skin, liver, intestine, spleen, brain, and lung.

**Table 1:** Study cohort description.

**Anatomic site**	**Age**	**Sex**	**Status**
**Pancreas**	Y: 23/35	4 M and 1 F	Normal
	M: 42/50	4 M and 1 F	Normal
	O: 69/76	4 M and 1 F	Normal
			
**Skin**	Y: 19/30	1 M and 4 F	Normal
	M: 41/46	2 M and 3 F	Normal
	O: 70/90	1 M and 4 F	Normal
			
**Kidney**	Y: 18/30	4 M and 1 F	Normal
	M: 41/50	2 M and 3 F	Normal
	O: 71/79	4 M and 1 F	Normal
			
**Liver**	Y: 17/30	2 M and 3 F	Normal
	M: 41/54	2 M and 3 F	Normal
	O: 70/84	3 M and 2 F	Normal
			
**Intestine (colon)**	Y: 18/26	4 M and 1 F	Normal
	M: 43/59	1 M and 4 F	Normal
	O: 71/77	2 M and 3 F	Normal
			
**Spleen**	Y: 16/30	5 M and 0 F	Normal
	M: 41/48	4 M and 1 F	Normal
	O: 70/ 83	2 M and 3 F	Normal
			
**Brain Cortex**	Y: 13/29	2 M and 3 F	Normal
	M: 40/55	5 M and 0 F	Normal
	O: 66/79	2 M and 3 F	Normal
			
**Lung**	Y: 17/32	3 M and 2 F	Normal
	M: 51 /55	2 M and 3 F	Normal
	O: 74/76	3 M and 2 F	Normal

**Figure 1:**
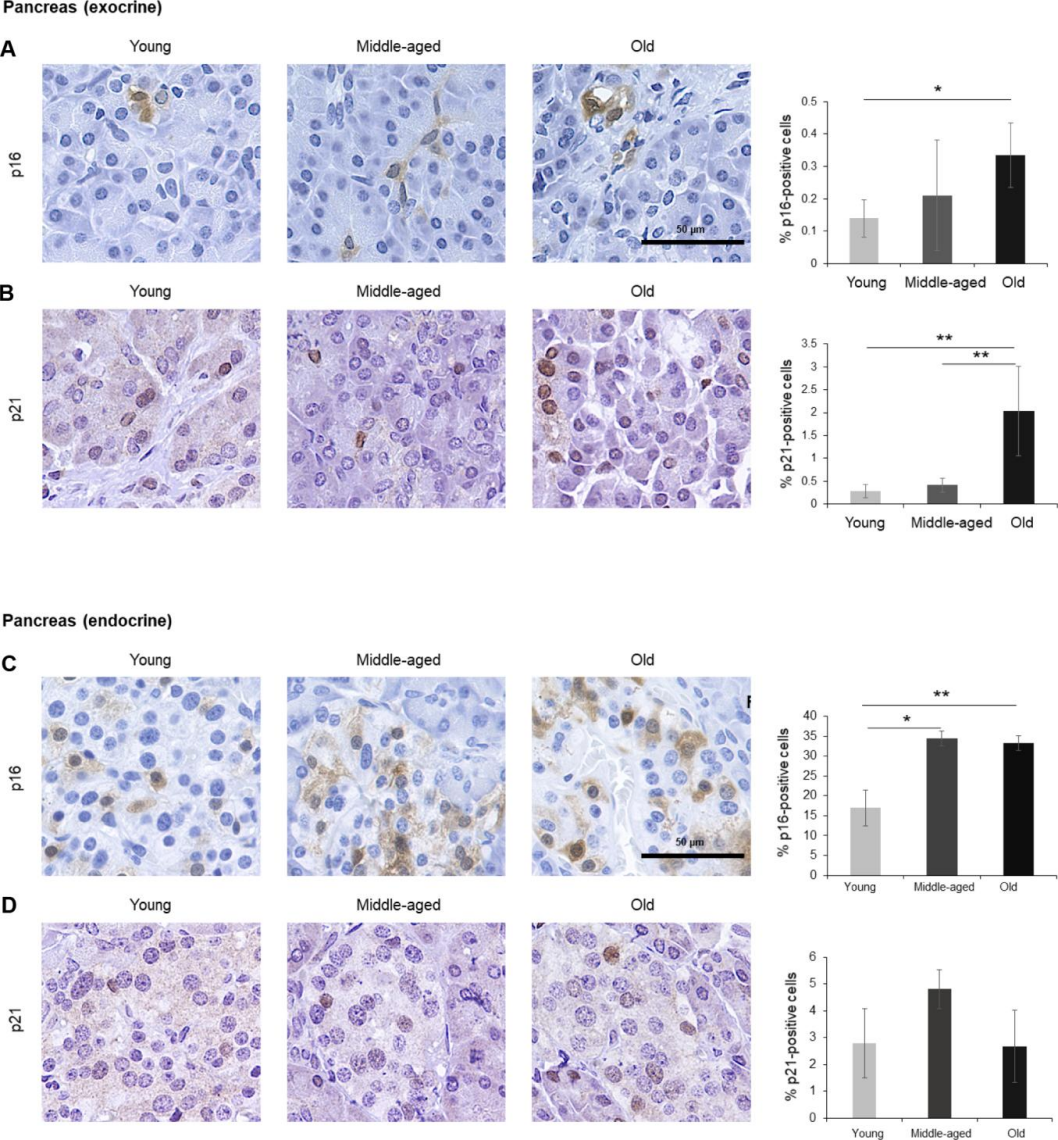
**Pancreas.** (**A**, **B**) Cells expressing p16 (**A**) or p21 (**B**) were identified by IHC staining in exocrine regions of the pancreas of Young, Middle-aged, and Old donors. (**C**, **D**) Cells expressing p16 (**C**) or p21 (**D**) were identified by IHC staining in the endocrine regions of the pancreas from Young, Middle-aged, and Old donors. Graphs represent the quantification (%) of p16-positive (**A**, **C**) and p21-positive (**B**, **D**) cells from 5 tissue cores from independent donors per organ and age group; data represent the means ±SD from 5 different donors. *p* values were determined by one-way ANOVA with Tukey adjustments for multiple comparisons where appropriate. **, *p* < 0.01; *, *p* < 0.05.

**Figure 2:**
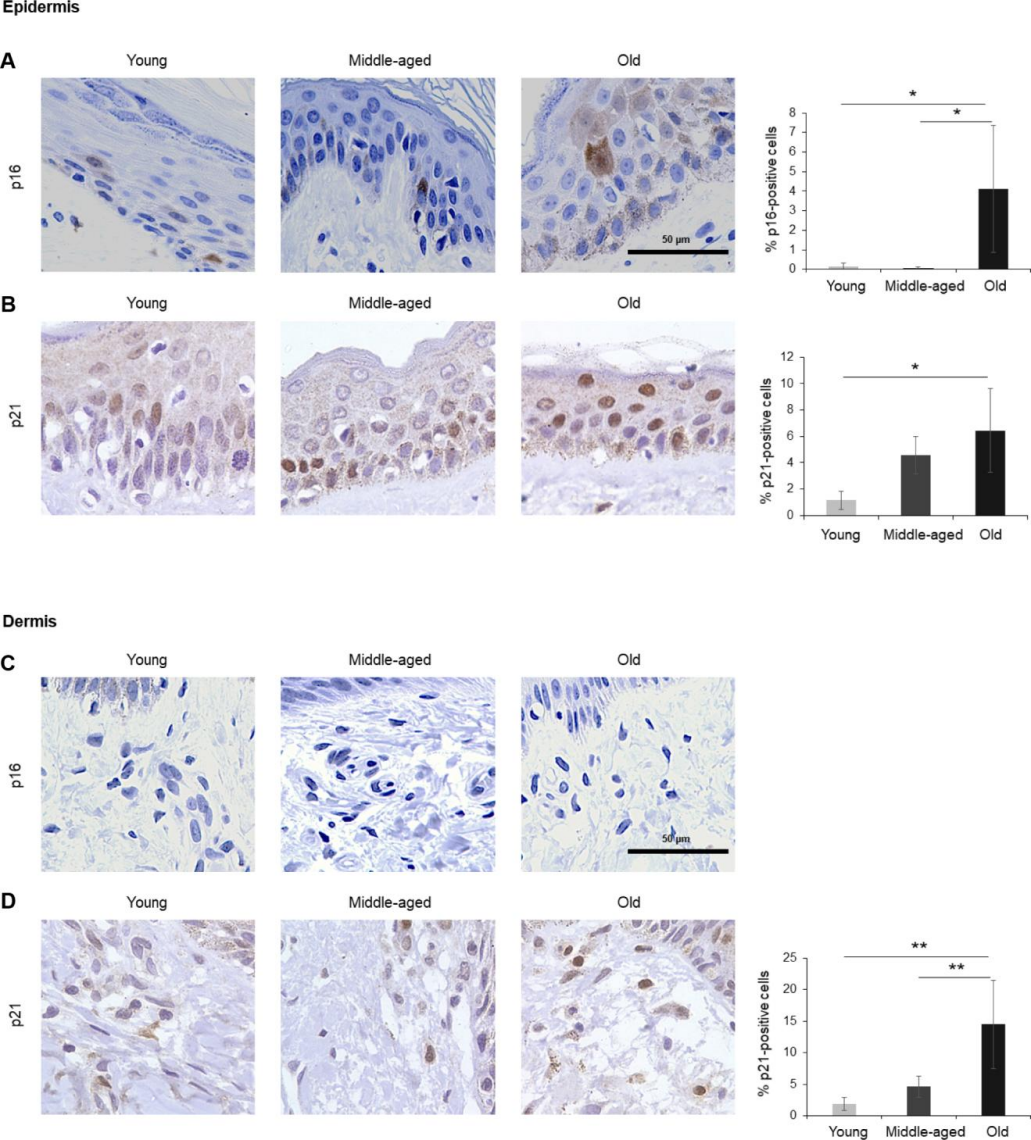
**Skin.** (**A**, **B**) Cells expressing p16 (**A**) or p21 (**B**) were identified by IHC staining in the epidermis of Young, Middle-aged, and Old donors. (**C**, **D**) Cells expressing p16 (**C**) or p21 (**D**) were identified by IHC staining in the dermis from Young, Middle-aged, and Old donors. Graphs represent the quantification (%) of p16-positive (**A**) and p21-positive (**B**, **D**) cells from 5 tissue cores from independent donors per organ and age group; data represent the means ±SD from 5 different donors. *p* values were determined by one-way ANOVA with Tukey adjustments for multiple comparisons where appropriate. **, *p* < 0.01; *, *p* < 0.05.

**Figure 3:**
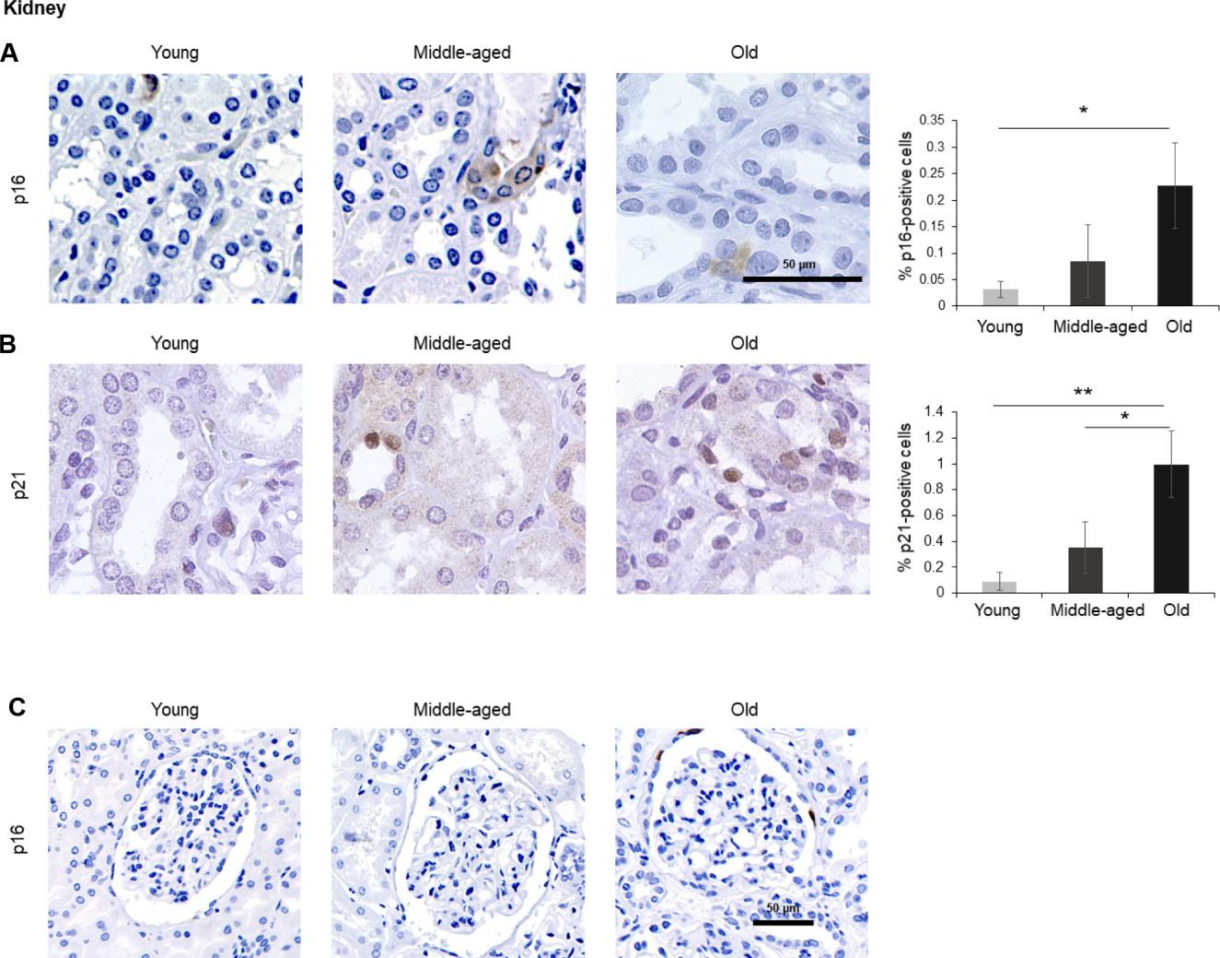
**Kidney.** (**A**, **B**) Cells expressing p16 (**A**) or p21 (**B**) were identified by IHC staining in the renal cortex of kidney of Young, Middle-aged, and Old donors. (**C**) Decreased magnification images showing p16-positive cells in the Bowman capsule of Old donors. Graphs represent the quantification (%) of p16-positive (**A**) and p21-positive (**B**) cells from 5 tissue cores from independent donors per organ and age group; data represent the means ±SD from 5 different donors. *p* values were determined by one-way ANOVA with Tukey adjustments for multiple comparisons where appropriate. **, *p* < 0.01; *, *p* < 0.05.

**Figure 4:**
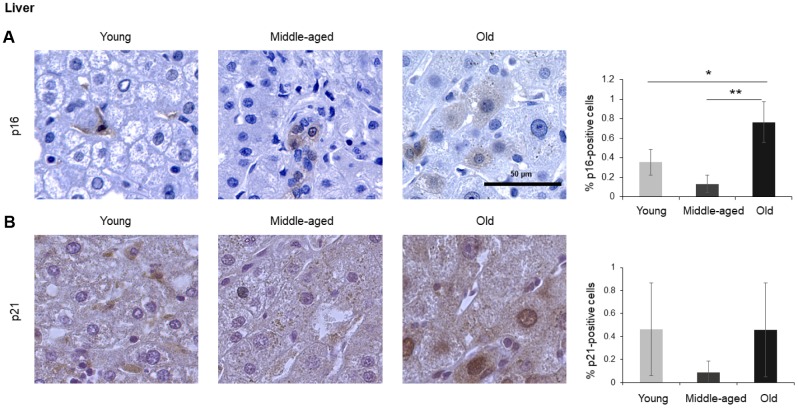
**Liver.** Cells expressing p16 (**A**) or p21 (**B**) were identified by IHC staining in the liver of Young, Middle-aged, and Old donors. Graphs represent the quantification (%) of p16-positive (**A**) and p21-positive (**B**) cells from 5 tissue cores from independent donors per organ and age group; data represent the means ±SD from 5 different donors. *p* values were determined by one-way ANOVA with Tukey adjustments for multiple comparisons where appropriate. **, *p* < 0.01; *, *p* < 0.05.

**Figure 5:**
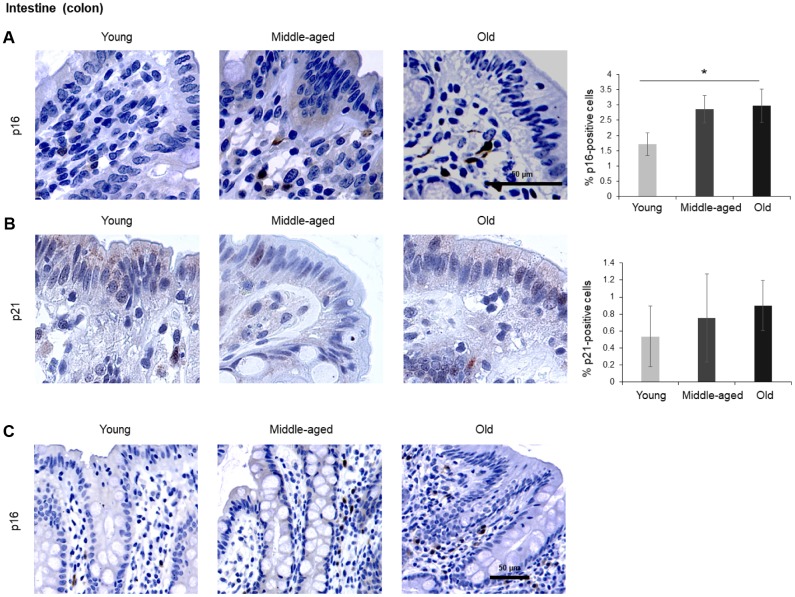
**Intestine (colon).** (**A**, **B**) Cells expressing p16 (**A**) or p21 (**B**) were identified by IHC staining in the colon of Young, Middle-aged, and Old donors. (**C**) p16-expressing cells in colon shown at lower magnification (20x). Graphs represent the quantification (%) of p16-positive (**A**) and p21-positive (**B**) cells from 5 tissue cores from independent donors per organ and age group; data represent the means ±SD from 5 different donors. *p* values were determined by one-way ANOVA with Tukey adjustments for multiple comparisons where appropriate. *, *p* < 0.05.

**Figure 6:**
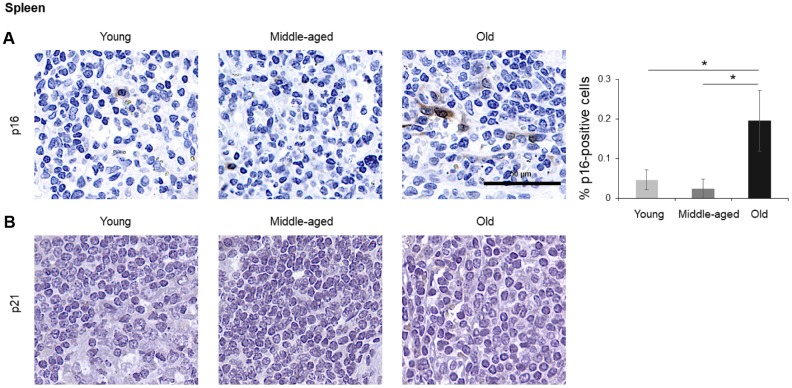
**Spleen.** Cells expressing p16 (**A**) or p21 (**B**) were identified by IHC staining in the spleen of Young, Middle-aged, and Old donors. Graph represents the quantification (%) of p16-positive cells from 5 tissue cores from independent donors per organ and age group; data represent the means ±SD from 5 different donors. *p* values were determined by one-way ANOVA with Tukey adjustments for multiple comparisons where appropriate. *, *p* < 0.05.

**Figure 7:**
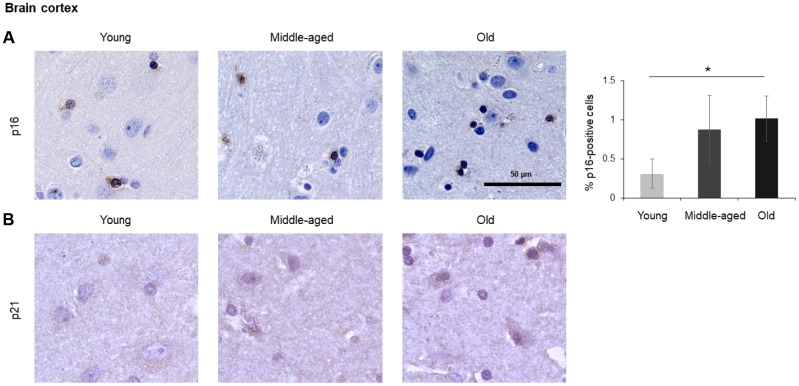
**Brain cortex.** Cells expressing p16 (**A**) or p21 (**B**) were identified by IHC staining in the brain cortex from Young, Middle-aged, and Old donors. Graph represents the quantification (%) of p16-positive cells from 5 tissue cores from independent donors per organ and age group; data represent the means ±SD from 5 different donors. *p* values were determined by one-way ANOVA with Tukey adjustments for multiple comparisons where appropriate. *, *p* < 0.05.

**Figure 8:**
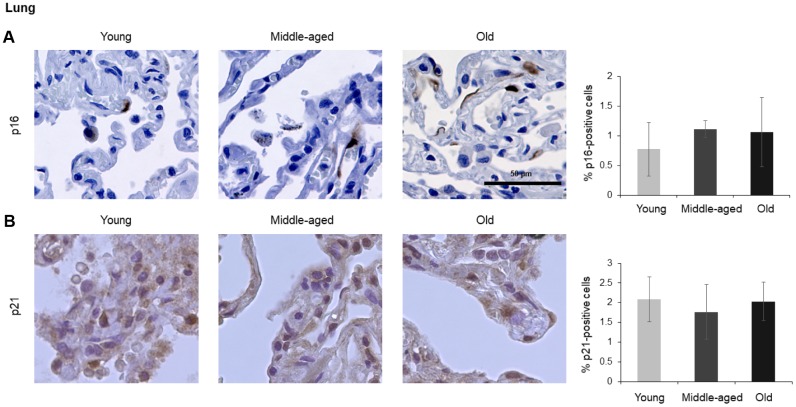
**Lung.** Cells expressing p16 (**A**) or p21 (**B**) were identified by IHC staining in the lung from Young, Middle-aged, and Old donors. Graph represents the quantification (%) of p16-positive cells (**A**) and p21-positive cells (**B**) from 5 tissue cores from independent donors per organ and age group; data represent the means ±SD from 5 different donors.

**Figure 9:**
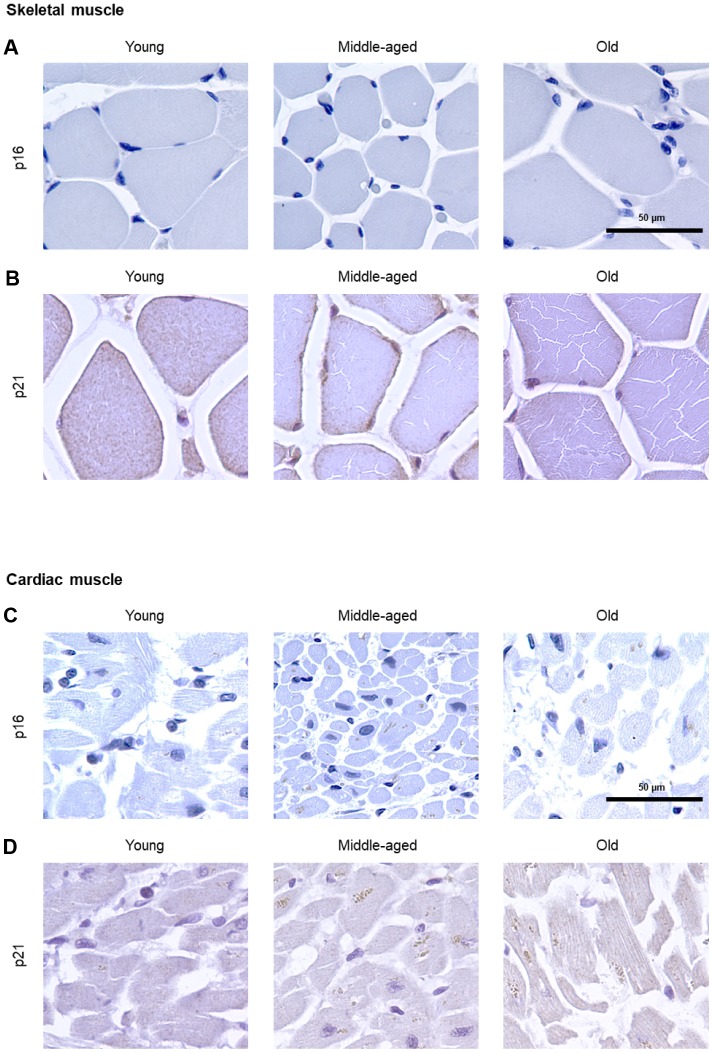
**Muscle.** IHC analysis to detect cells expressing p16 (**A**) or p21 (**B**) in skeletal muscle of Young, Middle-aged, and Old donors. IHC analysis to detect cells expressing p16 (**C**) or p21 (**D**) in cardiac muscle of Young, Middle-aged, and Old donors. These proteins were undetectable in these muscle biopsies (5 cores per organ and age group).

The numbers of cells expressing p21 and/or p16 differed among the different organs and age groups. In the exocrine pancreas, kidney, and epidermis we observed increased levels of both p16-positive cells and p21-positive cells with advancing age (Figures 1–3), even though the percentages of positive cells were low overall for both p16 and p21. In the dermis, p21-positive cells increased with age, comprising up to 15% of cells in the Old age group (Figure 2), while p16 was almost undetectable. By contrast, p16-positive cells increased with age in the endocrine pancreas, liver, intestine (colon), and brain cortex (Figures 1, 4–7), while p21 was low or unchanged. Interestingly, in the lung, cells positive for either p16 or p21 were detected in all age groups, but they did not increase in abundance with age (Figure 8), while in skeletal and cardiac muscle, we did not detect cells positive for either p16 or p21 (Figure 9).

### Pancreas

Cells in the exocrine pancreas, which produces factors required for digestion, displayed p16-positive staining in both the cytoplasm and the nucleus, although the percentage of p16-positive cells was very low (<0.5% in older donors) (Figure 1A). By contrast, p21 signals were mainly found in the nucleus and showed a stronger increase in the Old cohort, where up to 2% of cells expressed p21 (Figure 1B). The endocrine pancreas also included cells that expressed these markers (Figure 1C, 1D). In islets of Langerhans, p16-positive cells were found in Young donors and increased significantly with age, reaching up to 35% of cells in the Old group, while p21-positive cells were less abundant and did not show a significant rise with age. Diffuse p16-staining was identified in both nuclear and cytoplasmic compartments (Figure 1C), as observed in other tissues in our study (below). The age-dependent increase in senescent cells in the endocrine pancreas may be relevant to the impairment in glucose metabolism linked to diseases of the elderly such as diabetes and cardiovascular pathology [33–35], and may help explain the benefit of senolytic drugs in diabetes [35]. The increase in p16- or p21-positive cells, indicative of senescence, agrees with earlier studies showing an increase of senescent cells in the pancreas with age [36].

### Skin

The largest organ in the body, the skin covers and protects the entire external surface. We analyzed separately the two main skin layers, epidermis and dermis. In the epidermis, the outermost layer of the skin, cells positive for p16 or p21 increased with age, reaching 4% and 6% of total cells in Old, respectively (Figure 2A, 2B). As observed in other tissues in this study, p16 staining was observed in both the nucleus and cytoplasm of cells in the epidermis (Figure 2A), while p21 staining was mainly nuclear (Figure 2B). In the dermis, by contrast, we were unable to detect p16 (Figure 2C), and p21 increased with aging (Figure 2D). These results agree with previous reports showing that p16-positive cells accumulate in skin during aging and that a progressive accumulation of senescent keratinocytes and fibroblasts causes alterations in skin architecture and function, leading to tissue deterioration [37–39].

### Kidney

A small number of p16-positive cells increasing moderately with donors’ age (up to 0.2% of all cells) was observed in the kidney. Positive p16 staining was mainly present in the tubules and was distributed in both nucleus and cytoplasm (Figure 3A), although we identified a few p16-positive cells in the Bowman capsule of Old donors (Figure 3C). In addition, p21-positive cells also increased significantly with age (reaching up to 1% in the Old group); these cells were localized in renal tubules, and the signal for p21 was restricted to the nucleus (Figure 3B). These observations are in keeping with earlier reports [40].

### Liver

The liver is the biggest gland in the human body. It is composed of different cell types including hepatocytes (parenchymal cells), and non-parenchymal cells such as biliary epithelial cells, Kupffer cells, liver sinusoidal endothelial cells, hepatic stellate cells, and lymphocytes. We found that p16-positive and p21-positive cells were only <1% even in Old tissues (Figure 4), but p16-positive cells increased significantly during aging, in agreement with earlier studies [7]. Interestingly, while most p16-positive cells appeared to be non-parenchymal in Young donors, tissues from Old donors displayed a stronger p16-positive signal in hepatocytes (Figure 4). There was no significant change observed in cells staining positive for p21 (Figure 4B).

### Intestine

Given the impact of aging on gut dysfunction, we included intestinal tissues in our analysis. Among the three different compartments in the colon, the columnar epithelium and the intestinal glands (crypts of Lieberkuhn) did not show significant staining. However, the lamina propria (the connective tissue and inflammatory cells surrounding the crypts), stained positive for p16 in the cytoplasm, and these signals increased with age (Figure 5A). p21 showed nuclear staining and increased during aging, although it did not reach statistical significance (Figure 5B). An image of p16-positive cells at lower magnification (20x) is included (Figure 5C).

### Spleen, brain, lung, muscle

In the spleen, there were no p21-positive cells and very few p16-positive cells (<0.2% in Old), although the number of p16-positive cells increased significantly with advancing age. As in other tissues, p16 signals were evenly distributed between nucleus and cytoplasm (Figure 6). In the brain cortex, which undergoes major molecular and functional changes with aging, we did not detect p21-positive cells, but we observed p16- positive staining; these signals appeared to be found in glial cells but not neurons or endothelial cells, and increased with age (Figure 7). In the lung we found both p16- and p21-positive cells among pneumocytes and alveolar macrophages, but we did not observe increased numbers of positive cells with aging; of note, the antibody recognizing p21 produced nonspecific background in the lung (Figure 8). Finally, in skeletal and cardiac muscle, we did not detect p16- or p21-positive cells (Figure 9).

### Concluding remarks

The systematic survey of cells expressing p16 or p21, two classical senescence markers, revealed unexpectedly distinct patterns, summarized in Table 2. While some organs displayed an increase in both p16-positive and p21-positive cells with age [exocrine pancreas, epidermis, and kidney (Figures 1–3)], the relative numbers of cells expressing one or the other marker varied widely, sometimes by an order of magnitude, suggesting that the senescence markers p16 and p21 were not always co-expressed in cells. Further supporting this point, some organs only showed increased numbers of cells expressing p16 (endocrine pancreas, liver, intestine, spleen, brain cortex) or only p21 (dermis) with advancing age (Figures 1, 2, 4–7). While these findings suggest that different tissues may trigger different senescence programs, with preferential rise of one marker or another, our results support the notion that with advancing age, p16 appears to be a better marker to identify cells in older tissues, presumably senescent cells.

**Table 2:** Summary of the fold differences in p16-positive and p21-positive cells in middle-aged and old participants relative to young.

**Anatomic site**	**p16**			**p21**		
	**Young**	**Middle-aged**	**Old**	**Young**	**Middle-aged**	**Old**
**Pancreas, exocrine**	1	1.52	2.41	1	1.45	7.12
**Pancreas, endocrine**	1	2.03	1.96	1	1.73	0.96
**Skin, epidermis**	1	0.73	21.41	1	3.41	4.80
**Skin, dermis**	-	-	-	1	2.47	7.73
**Kidney**	1	2.67	7.21	1	3.89	10.97
**Liver**	1	0.37	2.16	1	0.19	0.99
**Intestine (colon)**	1	1.67	1.73	1	1.40	1.68
**Spleen**	1	0.50	4.17	-	-	-
**Brain Cortex**	1	2.81	3.27	-	-	-
**Lung**	1	1.43	1.37	1	0.85	0.98

(-): no positive cells found.

Yet in other organs, advancing age did not elevate the numbers of cells expressing either p16 or p21 [e.g., lung (Figure 8)], and in some cases, these markers were undetectable in all age groups [e.g., skeletal or cardiac muscle (Figure 9)], suggesting that senescence in these organs may be characterized by other protein expression programs. Alternatively, mechanisms to clear senescent cells from tissues, such as immune cells [41], may be more active in the removal of p16- and/or p21-expressing cells in these organs.

The current survey can be expanded in many informative directions. For instance, we were unable to test if p16- or p21-expressing cells were also positive for the widely assayed senescence marker SA-β-Gal, as this enzyme is inert in FFPE tissues and can only be detected in fresh samples. Frozen tissue arrays would be needed for the simultaneous analysis and colocalization of these senescent markers. In this regard, the current recommendation to identify senescent cells is to assess the joint presence of multiple markers (e.g., p16 and p21), markers of lysosomal activity (such as SA-β-Gal), and markers of secretion (SASP factors) [1].

Other expansions of this analysis could include identifying p16- and p21-positive cells in other organs and tissues not included here, such as gallbladder, vascular tissues, testis, immune cells, smooth muscle, etc. In addition, access to larger sets of samples would permit the analysis of senescent markers in specialized cell populations within given tissues and organs. Given that cells positive for senescent markers are few in most tissues, this expansion would be particularly valuable. Finally, the inclusion of pairs of healthy tissues and corresponding disease tissues (e.g., sarcopenic vs. normal muscle, cirrhotic vs. normal liver, etc.) might help identify senescent cells implicated in tissue pathology. This information might be particularly valuable given the growing recognition that disease diagnosis, etiology, progression, and treatment vary in older populations [42].

In closing, this systematic study of p16- and p21-expressing cells in different organs across age groups helps to set the stage for a more comprehensive understanding of the impact of senescence in aging health and disease. It may also guide the development of superior markers and approaches to study senescent cells, and down the road, it may lead to the discovery of therapies directed at senescent cells for intervention in aging and age-related diseases.

## MATERIALS AND METHODS

### Immunohistochemistry

Immunohistochemistry (IHC) was performed on formalin-fixed, paraffin-embedded (FFPE), custom-designed, human normal tissue arrays (Pantomics). The arrays included five tissues per organ type and age group divided into Young, Middle-aged, and Old donors (Table 1).

For p16, immunocytochemistry was performed according to the protocol provided by the CINtec for p16^INK4a^ Cytology Kit (Ventana) using the ready-to-use reagents from the kit. Briefly, after antigen retrieval with citrate solution, slides were rinsed and blocked with a peroxidase-blocking reagent, and incubated with p16^INK4a^ antibody (clone E6H4). Immunoreactive signals on slides were visualized with DAB Quanto chromogen (Thermo Scientific).

For p21 staining, antigen retrieval after deparaffinization was performed by microwaving in 1X Dako Target Retrieval Solution (Agilent) and then tissues were incubated with Ultravision Hydrogen Peroxide Block (Thermo Fisher). Slides were incubated with anti-p21 antibody (1:100; Abcam) at 4°C for 16 hours, and subsequently incubated with the Primary Antibody Amplifier Quanto and then with HRP Polymer Quanto (Thermo Fisher). Immunoreactive signal was visualized with DAB Quanto chromogen (Thermo Fisher).

Slides were subsequently counterstained with hematoxylin, dehydrated, mounted with permanent mounting medium, and covered with a coverslip. All steps, except for the epitope retrieval, were performed at 25 °C. Immunoreactivity was visualized by light microscopy.

### Slide scanning and image analysis of tissue arrays

Stained tissue sections were imaged at 20x and 40x total magnification using a KEYENCE All-In-One Fluorescence Microscope (BZ-X700). After IHC staining, images were analyzed using ImageJ. p16 and p21 were quantified using a color deconvolution algorithm to identify diaminobenzidine (DAB) and haematoxylin positivity in at least 3 defined ImageJ-based macros regions of interest (ROI) for each donor. Specific ROI were selected for each tissue microarray spot to exclude folded tissues and inappropriate tissue regions. Percentages of positive cells were calculated for each tissue and age group.
